# Impact of age and rurality on colorectal cancer outcomes in the United States

**DOI:** 10.1007/s10552-026-02150-3

**Published:** 2026-03-19

**Authors:** Caitlyn Grunert, Meng-Han Tsai, Charles R. Rogers, Sydney Howard, Rachel Hogg-Graham, Courtney Martin, Darwin Conwell, Adetunji T. Toriola, Avinash Bhakta, Justin X. Moore

**Affiliations:** 1 Center for Health, Engagement, & Transformation, Department of Behavioral Science, University of Kentucky, Lexington, KY USA; 2 Department of Health Management and Policy, College of Public Health, University of Kentucky, Lexington, USA; 3 Georgia Prevention Institute, Augusta University, Augusta, GA USA; 4 Georgia Cancer Center, Augusta University, Augusta, GA USA; 5 Men’s Health Inequities Research Lab, Milwaukee, WI USA; 6 Markey Cancer Center, University of Kentucky, Lexington, KY USA; 7 Department of Internal Medicine, College of Medicine, University of Kentucky, Lexington, KY USA; 8 Division of Public Health Sciences, Department of Surgery, and Siteman Cancer Center, Washington University School of Medicine, St. Louis, MO USA; 9 Department of Surgery, College of Medicine, University of Kentucky, Lexington, KY USA; 10 Population Science, Department of Internal Medicine, Community Impact, Markey Cancer Center, University of Kentucky College of Medicine, 473 Healthy Kentucky Research Building, 760 Press Avenue, Lexington, KY 40536-0679 USA

**Keywords:** Early-onset colorectal cance, Rural health disparities, Rural-urban differences, Late-stage diagnosis, Age at diagnosis

## Abstract

**Background:**

While overall colorectal cancer (CRC) rates have declined in the United States (U.S.), early-onset CRC (EO-CRC), diagnosed before age 50, has increased. Rural U.S. residents face higher CRC incidence than non-rural counterparts. We aimed to examine the relationship between age at diagnosis and EO-CRC outcomes and whether rurality modify these associations.

**Methods:**

We analyzed data from 835,907 patients aged 20–79 years in the 2006–2020 SEER Program. Rurality was defined using U.S. Department of Agriculture Rural–Urban Commuting Area (RUCA) codes. Multivariable logistic regression models were used to examine associations between age, rurality, and late-stage CRC diagnosis. Competing-risk regression models were applied to evaluate CRC-specific mortality, accounting for non–CRC deaths. Patients aged 50–59 years residing in non-rural areas served as the reference group for joint age–rurality analyses. All models adjusted for demographic, socioeconomic, clinical, and treatment factors.

**Results:**

Patients aged 30–39 (aOR: 1.49, 95% CI: 1.44–1.54) and 40–49 (aOR: 1.43, 95% CI: 1.40–1.46) had significantly higher odds of late-stage CRC compared with those aged 50–59, with the strongest associations observed among non-rural patients aged 30–39 (aOR: 1.52, 95% CI: 1.46–1.58). In competing-risk models, rural patients were at higher CRC-specific mortality across all age groups, with relative hazard increases ranging from 8% in older adults (70-79) to 21% in younger adults (20-29) compared with their non-rural counterparts.

**Conclusion:**

Adults aged 30–49 had higher odds of late-stage CRC than those 50–59, especially in non-rural areas. Rural residence was associated with higher CRC-specific mortality across all ages. Overall, age more strongly predicted late-stage diagnosis, while rurality more strongly predicted mortality. Earlier detection and improved clinical awareness in younger adults may reduce advanced-stage disease and CRC deaths.

**Supplementary Information:**

The online version contains supplementary material available at 10.1007/s10552-026-02150-3.

## Introduction

The incidence of early-onset colorectal cancer (EO-CRC), defined as CRC diagnosed at the age of 18–49, has been increasing over the past few decades [1]. Several factors have been proposed to explain this phenomenon, including a family history of CRC, inflammatory bowel disease (IBD), physical inactivity, tobacco use, elevated body mass index (BMI), diabetes, and unhealthy dietary patterns [2]. Although the American Cancer Society adjusted the recommended screening age from 50 to 45 after extensive analysis of disease risk, many young adults still fall below this threshold and therefore remain ineligible for routine screening, despite the rising incidence of EO-CRC in this population [3]. As a result, these young individuals may face delayed diagnosis, limited access to preventive care, and an increased likelihood of being diagnosed at an advanced stage that may contribute to higher mortality [4].

More importantly, patients living in rural areas consistently experience higher mortality rates for CRC than those living in urban areas [5], largely due to limited healthcare resources [6]. For example, patients living in rural areas often encounter restricted access to medical and oncology providers for cancer care [7]. This can lead to later-stage CRC diagnoses and reduced chances of receiving timely treatment and supportive care compared to those living in urban areas [4]. Additionally, rural patients tend to present with more advanced disease stages than their urban counterparts [8]. Building on this evidence, several studies have further shown that rurality is associated with lower rates of CRC screening and chemotherapy treatment following diagnosis [9, 10]. Age is also a key factor of CRC risk, with incidence nearly doubling every five years before age 50 and increasing by approximately 30% with each additional five-year interval after age 50 [11]. Moreover, between 2013 and 2017, states such as Kentucky, West Virginia, Arkansas, Mississippi, and Louisiana, all of which have large rural populations in the southern United States, reported age-adjusted CRC mortality rates among the highest in the country [12]. These patterns highlight persistent differences in both disease stage at diagnosis and adherence to treatment guidelines, with rural residents facing continued challenges in accessing timely and appropriate cancer care compared to their urban counterparts [13, 14].

While much of the existing research has focused on older, screening-eligible adults (aged 45 and older), emerging literature has begun to examine the growing burden of EO-CRC among younger populations. However, limited evidence explores how rural residence may influence the stage at diagnosis and cause-specific survival in this younger age group. To address this gap, the present study examined the associations between age at diagnosis and two key outcomes: late stage at diagnosis and cause-specific mortality among individuals with EO-CRC. We further evaluated whether these associations differed by rural versus non-rural residence. A clearer understanding of these patterns may help inform clinical practice by prioritizing prevention and treatment strategies tailored to younger adults, particularly those living in rural areas.

## Methods

### Data source

We conducted a secondary data analysis using retrospective cohort patient data from the.

Surveillance Epidemiology and End Results (SEER) 21 (previously 22) registries (http://seer.cancer.gov), November 2022 submission (2006–2022). This time frame was selected because 2022 represented the most recent year of available data at the time of the request. Since 1973, the SEER program has provided information regarding cancer statistics to reduce the cancer burden among the U.S. population, and the data are collected and curated by the National Cancer Institute Division of Cancer Control and Population Sciences. Specifically, the SEER 21 registry (previously 22) covers approximately 47.9% of the U.S. population (based on the 2020 census) including cancer patient data from 22 geographic areas and cancer registries [15].

## Ethical statement

This study was deemed exempt by the Institutional Review Boards of the University of Kentucky because we utilized pre-existing secondary data that are publicly available and de-identified.

## Study population

**Fig. 1:**
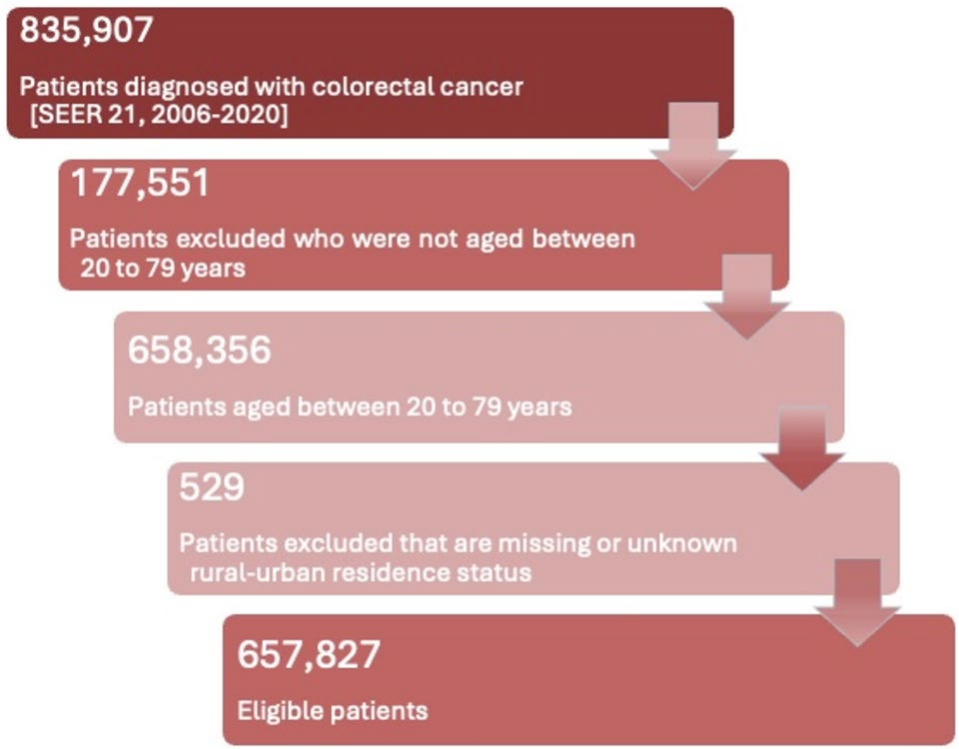
Flowchart of SEER eligible patients

Our study population included patients diagnosed with colorectal cancer (CRC) as defined by the SEER Site Recode ICD-O-3/WHO 2008 classification: colon cancer (C180–C189), rectosigmoid junction cancer (C199), and rectal cancer (C209) [16]. Data were obtained from the SEER 21 database using SEER*Stat software, yielding 835,907 CRC cases diagnosed between 2006 and 2020 with follow-up through 2022 [17]. We excluded individuals younger than 20 or older than 79 due to limited case counts and typical CRC screening guidelines ending around age 75 (*n* = 177,551). We also excluded those with missing or unknown rurality based on 2010 RUCA codes (n = 529). After applying these criteria, our final analytic sample included 657,827 individuals (Fig. 1). Additional exclusions for missing stage or treatment variables were applied only in outcome-specific analyses and were not used to define the overall analytic cohort. Stage at diagnosis, chemotherapy, surgery, radiation, and mortality variables were not excluded during analysis. Stage at diagnosis was operationalized to create a late-stage indicator (localized vs. regional/distant) and a categorical stage variable (localized, regional, distant, unknown). Treatment variables were categorized as “yes” or “no/unknown” for chemotherapy, “received” or “none/unknown/missing” for radiation, and “yes” or “none” for surgery. CRC-specific mortality was defined using cause-specific death classification and vital status to derive an event indicator for CRC death. Patients with unknown stage were retained in the analytic cohort but excluded in models where stage classification was required to define the outcome (e.g., late-stage diagnosis models excluded “unknown/unstaged”). Treatment receipt models used the “no/unknown” categories as the comparison group.

## Exposure(s) of interest

Our primary exposure of interest was age at diagnosis and categorized as deciles: (1) 20–29 years, (2) 30–39 years, (3) 40–49 years, (4) 50–59 years, (5) 60–69 years, and (6) 70–79 years. In addition, census tract rurality was our secondary exposure of interest with rural, non-rural designation for each patient. The first group is based on the U.S. Department of Agriculture (USDA)'s Rural Urban Commuting Area (RUCA) codes with two categories: Rural area commuting focused (codes 8, 9, 10) and non-rural area commuting focused (all other listed codes) [18].

## Outcome(s) of interest

There were two primary outcomes of interest in this study: (1) stage at diagnosis based on the SEER summary stage variable and categorized as late-stage (if regional or distant) or early stage (if in situ and localized) and (2) causes-specific death for CRC. Secondary outcomes of interest included in supplementary results are (1) initial surgical treatment given or not, regardless of reason; (2) initial radiation treatment given or not, regardless of reason; and (3) initial chemotherapy treatment given or not, regardless of the reason.

## Covariate(s) of interest

Clinical characteristics included tumor stage, grade, and primary site. Tumor stage was categorized as localized, regional, distant, or unknown. Tumor grade was categorized as Grade I (well-differentiated), Grade II (moderately differentiated), Grade III (poorly differentiated), Grade IV (undifferentiated), and unknown grade was retained as a separate category. Primary tumor site was dichotomized as right-sided or left-sided colorectal cancer. Right-sided tumors included cancers of the cecum (C18.0), appendix (C18.1), ascending colon (C18.2), hepatic flexure (C18.3), and transverse colon (C18.4). Left-sided tumors included cancers of the splenic flexure (C18.5), descending colon (C18.6), sigmoid colon (C18.7), overlapping lesions of the colon (C18.8), colon not otherwise specified (C18.9), rectosigmoid junction (C19.9), and rectum (C20.9). Year of diagnosis was grouped into three intervals (2006–2010, 2011–2015, and 2016–2020) to account for temporal changes in colorectal cancer (CRC) detection and treatment. Chemotherapy was dichotomized as yes and no/unknown. Radiation therapy was categorized as received versus none/unknown/missing. Surgical treatment was categorized as yes versus no. All covariates were included to control for potential confounding in analyses examining the associations between age at diagnosis, rurality, late-stage diagnosis, treatment receipt, and CRC-specific survival.

## Statistical analysis

We provide descriptive statistics, including means and standard deviations for continuous variables and frequencies with percentages for categorical variables, stratified by age group. These characteristics included rurality, patient demographics, tumor features, and initial treatment modalities. Bivariate analyses were conducted to assess differences in these variables by age at diagnosis and rurality. Chi-square tests were used for categorical variables, and analysis of variance was used for continuous variables. Survival time was defined as months from date of diagnosis to death due to CRC, with deaths from other causes treated as censored observations.

We conducted sequential multivariable regression analyses (logistic and Cox proportional hazards models) to evaluate associations between age at diagnosis, rurality, and outcomes (late-stage diagnosis, initial surgical treatment, chemotherapy, radiation therapy, and CRC survival). For analyses requiring complete information on stage or treatment, individuals with missing or unknown values were excluded only from the corresponding model. Effect modification by rurality was assessed through inclusion of multiplicative interaction terms between age group and rural residence, with statistical significance evaluated using Wald tests (α = 0.05).

In primary models, patients aged 50–59 years served as the reference group, as this is the most commonly diagnosed age group and diagnoses after age 50 are not considered early-onset. All models were adjusted for age, race and ethnicity, marital status, sex, primary tumor site, rural, persistent poverty, and diagnosis year. Stratified analyses were also conducted by rurality, and rurality was not included as a covariate in stratified models. Secondary analyses examining treatment receipt by age group were performed and are presented in the Supplementary Materials. All results are reported as adjusted odds ratios (aORs) or hazard ratios (aHRs) with 95% confidence intervals (CIs). Analyses were conducted using SAS version 9.4 (SAS Institute Inc., Cary, NC).

To address competing risks and age-related heterogeneity in mortality, we conducted competing-risk analyses using Fine–Gray proportional sub-distribution hazard models (SHR). Non–CRC deaths were treated as competing events, and individuals alive at last follow-up were censored. To directly evaluate the joint effects of age and rurality, we constructed a combined age–rurality exposure variable and used adults aged 50–59 years residing in non-rural areas as the reference group. Age was examined in the following categories: 20–29, 30–39, 40–49, 50–59, 60–69, and 70–79 years, allowing for direct comparison of CRC-specific mortality risk across age and residence strata. Models were fit sequentially to assess the robustness of associations: Model 1 adjusted for rurality and diagnosis year to account for temporal trends; Model 2 additionally adjusted for sociodemographic characteristics including race, marital status, sex, and persistent poverty; and Model 3 further adjusted for clinical and treatment-related factors, including primary tumor site, stage at diagnosis, surgery, radiation therapy, and chemotherapy. Results are presented as SHRs with 95% confidence intervals, with age- and rurality-specific estimates reported in Supplemental Table 5**.**

## Results

### Sociodemographic characteristics of SEER patients 2006–2020

In Table 1, the majority of patients resided in non-rural areas, with 86.2% living in non-rural settings compared to 13.8% in rural areas. Male patients made up the majority overall (55.2%) and have higher representation within each respective age group, except 20–29 years. NH-White patients accounted for more than half of the sample, followed by Hispanic patients across all age groups. Married patients accounted for 44.3% of the sample, with the highest percentage residing in those aged 40–49 years. Although many patients (89.5%) resided in non-persistent poverty areas, a significant portion of those living in persistent poverty (12.4%) were in the 20–29 age group. The second largest age group living in persistent poverty was 50–69-year-olds (11.5%). Late-stage diagnosis increased with age until 40–49 years (49.7%) and then slightly declined (50–59 years: 43.7%; 60–69 years: 43.7%; 70–79 years: 42.7%). Further, left-sided CRC was more common in younger patients, with the highest proportion in the age of 40–49 (72.3%). Most notably, between 2016 and 2020, across all age groups, diagnosis decreased as age increased.

**Table 1:** Patient socio-demographics and other characteristics by age of diagnosis, among 657,931 CRC patients diagnosed SEER Years 2006 – 2020

	Total (*n* = 657,827)	20–29 years (*n* = 6,038, 0.9%)		30–39 years (*n* = 21,069, 3.2%)	40–49 years (*n* = 67,856, 10.4%)	50–59 years (*n* = 165,364, 25.1%)	60–69 years (*n* = 205,027, 31.2%)	70–79 years (*n* = 192,577, 29.3%)
Survival months, mean (SD)		55.2 (48.1)		57.5 (49.6)	61.1 (51.1)	61.8 (50.9)	56.4 (49.4)	49.7 (47.1)
Rurality								
Yes	90,557 (13.8%)	621 (10.3%)		2,187 (10.4%)	7,666 (11.3%)	21,011 (12.7%)	29,787 (14.5%)	29,285 (15.2%)
No	567,374 (86.2%)	5,417 (89.7%)		18,882 (89.6%)	60,190 (88.7%)	144,353 (87.3%)	175,240 (85.5%)	163,292 (84.8%)
Demographic characteristics								
Gender								
Male	363,348 (55.2%)	2,957 (48.9%)		10,820 (51.4%)	36,285 (53.5%)	93,647 (56.6%)	117,896 (57.5%)	101,743 (52.8%)
Female	294,583 (44.8%)	3,081 (51.0%)		10,249 (48.6%)	31,571 (46.5%)	71,717 (43.4%)	87,131 (42.5%)	90,834 (47.2%)
Race								
Hispanic	99,745 (15.2%)	1,543 (25.6%)		5,190 (24.6%)	13,571 (20.0%)	27,663 (16.7%)	29,393 (14.3%)	22,385 (11.6%)
NH-Asian	47,713 (7.3%)	468 (7.8%)		1,782 (8.5%)	5,800 (8.6%)	12,967 (7.8%)	14,720 (7.2%)	11,976 (6.2%)
NH-black	86,294 (13.1%)	649 (10.7%)		2,678 (12.7%)	9,754 (14.4%)	25,171 (15.2%)	27,513 (13.4%)	20,529 (10.7%)
NH-white	416,804 (63.4%)	3,261 (54.0%)		11,084 (52.6%)	37,817 (55.7%)	97,189 (58.8%)	13,1249 (64%)	136,204 (70.7%)
Other race	7,375 (1.1%)	117 (1.9%)		335 (1.6%)	914 (1.4%)	2,374 (1.4%)	2,152 (1.1%)	1,483 (0.8%)
Marital status								
Single	100,852 (15.3%)	3,221 (53.4%)		5,831 (27.7%)	13,948 (20.6%)	29,614 (17.9%)	29,215 (14.3%)	19,023 (9.9%)
Married	291,515 (44.3%)	1,268 (21.0%)		8,853 (42.0%)	31,347 (46.2%)	74,421 (45%)	91,702 (44.7%)	83,924 (43.6%)
Other^1^	107,980 (16.4%)	116 (1.9%)		1,100 (5.2%)	5,944 (8.8%)	19,696 (11.9%)	33,696 (16.4%)	47,428 (24.6%)
Unknown	157,584 (23.9%)	1,433 (23.7%)		5,285 (25.1%)	16,617 (24.5%)	41,633 (25.2%)	50,414 (24.6%)	42,202 (21.9%)
Persistent poverty status								
Persistent poverty area	87,817 (10.5%)	749 (12.4%)		2,366 (11.3%)	7,372 (10.9%)	18,964 (11.5%)	23,676 (11.5%)	19,759 (10.3%)
Non-persistent poverty area	747,561 (89.5%)	5,289 (87.6%)		18,703 (88.7%)	60,484 (89.1%)	146,400 (88.5%)	181,351 (88.5%)	172,818 (89.7%)
Stage								
Localized	202,007 (30.7%)	1,855 (30.7%)		4,829 (22.9%)	17,008 (25.1%)	52,032 (31.5%)	63,448 (30.9%)	62,835 (32.6%)
Late stage^2^	290,222 (44.1%)		2,305 (38.2%)	10,141 (48.1%)	33,693 (49.7%)	72,335 (43.7%)	89,496 (43.7%)	82,252 (42.7%)
Unknown	165,702 (25.2%)		1,878 (31.1%)	6,099 (28.9%)	17,155 (25.3%)	40,997 (24.8%)	52,083 (25.4%)	47,490 (24.7%)
Year of CRC diagnosis								
2006–2010	214,067 (32.5%)		1,465 (24.3%)	5,792 (27.5%)	21,761 (32.1%)	52,314 (31.6%)	64,248 (31.3%)	68,487 (35.6%)
2011–2015	216,717 (32.9%)		1,959 (32.4%)	6,807 (32.3%)	22,215 (32.7%)	55,958 (33.8%)	68,431 (33.4%)	61,347 (31.9%)
2016–2020	227,147 (34.5%)		2,614 (43.3%)	8,470 (40.2%)	23,880 (35.2%)	57,092 (34.5%)	72,348 (35.3%)	62,743 (32.6%)
Primary site								
Left	414,202 (62.9%)		3,168 (52.5%)	14,328 (68%)	49,079 (72.3%)	117,337 (70.9%)	127,849 (62.4%)	102,441 (53.2%)
Right	243,729 (37.1%)		2,870 (47.5%)	6,741 (32%)	18,777 (27.7%)	48,027 (29.1%)	77,178 (37.6%)	90,136 (46.8%)
Receipt of chemotherapy								
Yes	271,162 (41.2%)		2,457 (40.7%)	11,692 (55.5%)	37,803 (55.7%)	75,448 (45.6%)	84,087 (41.0%)	59,675 (30.9%)
No/Unknown	386,769 (58.8%)		3,581 (59.3%)	9,377 (44.5%)	30,053 (44.3%)	89,916 (54.4%)	120,940 (58.9%)	132,902 (69.0%)
Receipt of radiation								
Yes	77,803 (11.8%)		594 (9.8%)	3,187 (15.1%)	11,291(16.6%)	22,594 (13.7%)	23,778 (11.6%)	16,359 (8.5%)
No/Unknown	580,128 (88.2%)		5,444 (90.2%)	17,882 (84.9%)	56,565 (83.4%)	142,770 (86.3%)	181,249 (88.4%)	176,218 (91.5%)
Receipt of surgery								
Yes	530,129 (88.2%)		5,081 (84.2%)	17,134 (81.3%)	54,711 (80.6%)	134,208 (81.2%)	164,682 (80.3%)	154,313 (80.1%)
No/Unknown	127,802 (19.4%)		957 (15.8%)	3,935 (18.7%)	13,145 (19.4%)	31,156 (18.8%)	40,345 (19.7%)	38,264 (19.9%)
Cause of death								
Alive	377,540 (57.4%)		4,601 (76.2%)	15,032 (71.3%)	45,557 (67.1%)	109,570 (66.3%)	118,353 (57.7%)	84,427 (43.8%)
CRC-related	173,416 (26.3%)		1,232 (20.4%)	5,045 (24.0%)	18,005 (26.6%)	40,330 (24.4%)	53,950 (26.3%)	54,854 (28.5%)
Other (non-CRC)	106,975 (16.3%)		205 (3.4%)	992 (4.7%)	4,294 (6.3%)	15,464 (9.3%)	32,724 (16.0%)	53,296 (27.7%)

^1^ Divorced, Separated, or Widowed

*NH* non-hispanic

^2^ Distant and Regionalized

## Odds for late-stage diagnosis

In Table 2, when adjusted for age, race, marital status, sex, rurality, primary site, persistent poverty, and diagnosis year, patients aged 30–39 years (adjusted OR: 1.49, 95% CI 1. – 1.54) and 40–49 years (aOR: 1.43, 95% CI 1.40 – 1.46) were at an increased odds of late-stage CRC diagnosis, when compared to 50–59 years old at diagnosis. However, 20–29 years (aOR: 0.84, 95% CI 0.79 – 0.89) were observed to have reduced odds of late-stage CRC diagnosis among the overall population, compared to 50–59 years at the age of diagnosis. Stratifying for rurality (yes) and in fully adjusted models  (Table 2), rural patients aged 30–39 years (aOR: 1.30, 95% CI 1.17 – 1.45) and 40–49 years (aOR: 1.33, 95% CI 1.25 – 1.42) had an increased odds of late-stage CRC diagnosis compared to rural patients aged 50–59 years. However, rural patients aged 20–29 (aOR: 0.82, 95% CI 0.68 – 0.99) had reduced odds of late-stage CRC diagnosis compared to rural patients aged 50–59 years. In adjusted models among non-rural patients (Table 2), those aged 30–39 years (aOR: 1.52, 95% CI 1.46 – 1.58) and 40–49 years (aOR: 1.4, 95% CI 1.41 – 1.48) had increased odds of late-stage CRC diagnosis compared to non-rural patients aged 50–59 years. Further, patients aged 20–29 years (aOR: 0.84, 95% CI 0.79 – 0.90) had reduced odds of late-stage CRC diagnosis compared to non-rural patients aged 50–59 years.

**Table 2:** Odds of Late-Stage Diagnosis for Age of Onset CRC by Age Categories, Overall and Stratified by Rural, Non-Rural Status. SEER Patients, 2006–2020

	No. (%) of late stage diagnosed cases	No. of total cases	Unadjusted OR (95% CI)	Adjusted OR (95% CI)
Overall				
Aged 20 – 29 years	2,305 (38.2%)	6,038	0.89 (0.84, 0.95)	0.84 (0.79, 0.89)
Aged 30 – 39 years	10,141 (48.1%)	21,069	1.51 (1.46, 1.57)	1.49 (1.44, 1.54)
Aged 40 – 49 years	33,693 (49.7%)	67,856	1.43 (1.39, 1.46)	1.43 (1.40, 1.46)
Aged 50 – 59 years	72,335 (43.7%)	165,364	1.00 (Referent)	1.00 (Referent)
Aged 60 – 69 years	89,496 (43.7%)	205,027	1.02 (0.99, 1.03)	1.01 (0.99, 1.02)
Aged 70 – 79 years	82,252 (42.7%)	192,577	0.94 (0.93, 0.96)	0.93 (0.92, 0.95)
Among rural patients				
Aged 20 – 29 years	247 (56.3%)	621	0.87 (0.72, 1.05)	0.82 (0.68, 0.99)
Aged 30 – 39 years	1,047 (66.2%)	2,187	1.32 (1.19, 1.48)	1.30 (1.17, 1.45)
Aged 40 – 49 years	3,818 (66.3%)	7,666	1.33 (1.25, 1.42)	1.33 (1.25, 1.42)
Aged 50 – 59 years	9,540 (59.7%)	21,011	1.00 (Referent)	1.00 (Referent)
Aged 60 – 69 years	12,967 (58.3%)	29,787	0.94 (0.91, 0.98)	0.94 (0.90, 0.98)
Aged 70 – 79 years	12,529 (56.9%)	29,285	0.89 (0.86, 0.93)	0.89 (0.85, 0.93)
Among non-rural patients				
Aged 20 – 29 years	2,058 (55.3%)	5,417	0.90 (0.84, 0.96)	0.84 (0.79, 0.90)
Aged 30 – 39 years	9,094 (67.9%)	18,882	1.54 (1.48, 1.59)	1.52 (1.46, 1.58)
Aged 40 – 49 years	29.875 (66.5%)	60,190	1.44 (1.41, 1.47)	1.44 (1.41, 1.48)
Aged 50 – 59 years	62,795 (57.9%)	144,353	1.00 (Referent)	1.00 (Referent)
Aged 60 – 69 years	76,529 (58.6%)	175,240	1.03 (1.01, 1.04)	1.02 (1.00, 1.04)
Aged 70 – 79 years	69,723 (56.7%)	163,292	0.95 (0.93, 0.97)	0.94 (0.92, 0.95)
*P* value for multiplicative interaction			0.0008	0.06

Models adjusted for age, race, marital status, sex, rurality*, primary site, persistent poverty, and diagnosis year.

^*^Stratified models are not adjusted for rurality

## Hazard of colorectal cancer death

When adjusted for age, race, marital status, sex, rurality, stage, primary site, persistent poverty, and diagnosis year (Table 3), patients aged 20–29 years (aHR: 0.96, 95% CI: 0.91–1.02), 30–39 years (aHR: 0.95, 95% CI: 0.93–0.98), and 40–49 years (aHR: 0.94, 95% CI: 0.92–0.96) had a reduced hazard of CRC death compared to patients aged 50–59 years. When stratified by rurality (yes) (Table 3), rural patients aged 40–49 years (aHR: 0.92, 95% CI: 0.88–0.96) had a lower hazard of CRC death compared to rural patients aged 50–59 years. Similarly, among non-rural patients, those aged 30–39 years (aHR: 0.94, 95% CI: 0.92–0.97), and 40–49 years (aHR: 0.94, 95% CI: 0.93–0.96) had a reduced hazard of CRC death compared to non-rural patients aged 50–59 years.

**Table 3:** Multivariable Hazard Ratios (HR) and Associated 95% Confidence Intervals for Risk of CRC Death Estimated Using Cox Proportional Hazard Model. SEER Patients, 2006 – 2020

	No. (%) of CRC deaths	Mean survival time (SE)	Unadjusted HR (95% CI)	Adjusted HR (95% CI)
Overall				
Aged 20 – 29 years	1,319 (21.8%)	125.41 (0.97)	0.87 (0.82, 0.92)	0.96 (0.91, 1.02)
Aged 30 – 39 years	5,408 (25.7%)	123.23 (0.55)	1.01 (0.98, 1.04)	0.95 (0.93, 0.98)
Aged 40 – 49 years	19,380 (28.6%)	122.08 (0.31)	1.09 (1.07, 1.10)	0.94 (0.92, 0.96)
Aged 50 – 59 years	43,951 (26.6%)	126.59 (0.19)	1.00 (Referent)	1.00 (Referent)
Aged 60 – 69 years	59,791 (29.2%)	123.29 (0.19)	1.16 (1.14, 1.17)	1.12 (1.11, 1.14)
Aged 70 – 79 years	62,144 (32.3%)	115.15 (0.21)	1.38 (1.36, 1.39)	1.33 (1.32, 1.35)
Among rural patients				
Aged 20 – 29 years	152 (24.5%)	76.17 (1.56)	0.85 (0.72, 1.00)	1.04 (0.89, 1.23)
Aged 30 – 39 years	594 (27.2%)	92.73 (1.18)	0.94 (0.87, 1.03)	1.03 (0.95, 1.12)
Aged 40 – 49 years	2,404 (31.4%)	117.77 (0.92)	1.05 (0.99, 1.10)	0.92 (0.88, 0.96)
Aged 50 – 59 years	6,323 (30.1%)	120.95 (0.56)	1.00 (Referent)	1.00 (Referent)
Aged 60 – 69 years	9,261 (31.1%)	116.48 (0.48)	1.09 (1.06, 1.13)	1.14 (1.11, 1.18)
Aged 70 – 79 years	9,932 (33.9%)	110.09 (0.52)	1.30 (1.25, 1.33)	1.34 (1.30, 1.39)
Among non-rural patients				
Aged 20 – 29 years	1,167 (21.5%)	125.83 (1.02)	0.88 (0.83, 0.93)	0.95 (0.90, 1.01)
Aged 30 – 39 years	4,814 (25.5%)	123.54 (0.58)	1.02 (0.99, 1.10)	0.94 (0.92, 0.97)
Aged 40 – 49 years	16,976 (28.2%)	121.39 (0.32)	1.09 (1.07, 1.11)	0.94 (0.93, 0.96)
Aged 50 – 59 years	37,628 (26.1%)	127.42 (0.21)	1.00 (Referent)	1.00 (Referent)
Aged 60 – 69 years	50,530 (28.8%)	123.87 (0.20)	1.16 (1.15, 1.18)	1.12 (1.11, 1.14)
Aged 70 – 79 years	52,212 (31.9%)	115.68 (0.22)	1.39 (1.37, 1.41)	1.33 (1.31, 1.35)
*P* value for multiplicative interaction			< 0.0001	0.12

Models adjusted for age, race, marital status, sex, rurality*, stage, primary site, persistent poverty, and diagnosis year

^*^Stratified models are not adjusted for rurality

## Secondary analyses of treatment related outcomes

We conducted secondary analyses to examine the odds of receiving chemotherapy, radiation therapy, and surgical treatment by age group (Supplemental Tables 1–3). We then performed competing-risk analyses of CRC-specific mortality overall (Supplemental Table 4) and stratified by joint age and rurality categories using adults aged 50–59 living in non-rural areas as the reference group (Supplemental Table 5).

## Fine and gray competing risk analysis

Across all age strata, residence in rural areas was associated with a higher risk of CRC–specific mortality after accounting for all-cause competing risk. These associations were generally strongest in fully adjusted models that included sociodemographic, clinical, and treatment-related factors (Supplemental Table 4). Notably, individuals aged 20–29 years residing in rural areas exhibited the highest relative risk of CRC mortality compared with their non-rural counterparts (SHR: 1.21, 95% CI: 1.02–1.45). Increased risks were also observed among rural residents aged 30–49 years, with associations persisting after adjustment for diagnosis year, persistent poverty, and treatment receipt. In contrast, among individuals aged 50–79 years, rural, non-rural differences in CRC mortality were attenuated in fully adjusted models compared with partially adjusted models, although several associations remained statistically significant. Overall, these findings indicate that rural, non-rural differences in CRC–specific mortality persist across age groups, particularly among younger adults, even after accounting for competing risks and extensive covariate adjustment.

## Joint effects of age and rurality on late-stage diagnosis and CRC-specific mortality

In analyses examining both age and rurality, using adults aged 50–59 living in non-rural areas as the reference group, age emerged as a stronger predictor of late-stage diagnosis and CRC-specific mortality (Supplemental Table 5). Across late-stage models, the odds of being diagnosed at a later stage were generally similar between rural and non-rural residents within age strata; however, individuals aged 30–39 living in non-rural areas consistently exhibited a higher likelihood of late-stage CRC compared with their rural counterparts across models (Model 1 aOR: 1.54, 95% CI: 1.48–1.60 vs. aOR: 1.42, 95% CI: 1.28–1.58; Model 2 aOR: 1.52, 95% CI: 1.46–1.58 vs. aOR: 1.43, 95% CI: 1.28–1.58; and Model 3 aOR: 1.25, 95% CI: 1.19–1.31 vs. aOR: 1.12, 95% CI: 0.99–1.27). In contrast, competing-risk models accounting for non–CRC mortality demonstrated an opposing pattern, with rural residence associated with a higher risk of CRC-specific mortality across all age strata, though non-significant effects in those aged <39 years. After adjustment for sociodemographic, clinical, and treatment-related factors, individuals aged 40–49 exhibited an 11% increased risk of CRC-related death relative to the reference group (SHR: 1.11, 95% CI: 1.06–1.15), supporting the conclusion that age plays a prominent role in CRC mortality risk.

## Discussion

### Key findings and interpretations

This study revealed important age-related differences in stage at diagnosis, mortality, and treatment patterns among patients with colorectal cancer (CRC), with some variation by rural versus non-rural residence. Patients diagnosed at a younger age (20–29 years) were 11% and significantly less likely to present with late-stage disease compared to those aged 50–59, a pattern observed in both rural and non-rural populations, though non-significant in rurality stratified models. In contrast, individuals aged 30–39 and 40–49 years had higher odds of late-stage diagnosis across the full sample, with these associations persisting in both rural and non-rural settings. Non-rural patients in these age groups generally had slightly higher odds than their rural counterparts. Although statistically significant interactions between age and rurality were observed for both late-stage diagnosis and CRC-specific mortality, the magnitude and direction of age-related associations were largely consistent across rural and non-rural strata. Individuals aged 30–49 had a higher risk of late-stage CRC diagnosis, despite lower overall mortality in younger patients.

To further evaluate whether age or rurality more strongly influenced CRC outcomes, we conducted joint age–rural analyses using adults aged 50–59 living in non-rural areas as the reference group. These analyses demonstrated that age, rather than rural residence, was the dominant predictor of late-stage diagnosis. Across models, individuals aged 30–39 exhibited significantly higher odds of late-stage CRC regardless of rural or non-rural residence.

Stratified competing-risk analyses showed rural residence remained associated with higher CRC-specific mortality across age groups, even after adjusting for diagnosis year, sociodemographic, clinical factors, and treatment. Notably, individuals aged 20–29 had the highest risk of CRC-specific mortality in the fully adjusted model. Mortality risk remained lower for younger patients overall, the consistent increase in late-stage diagnosis among those aged 30–49 highlights a concerning trend. Stratified analyses confirmed these patterns and showed that younger patients across all age groups were more likely to receive chemotherapy, radiation, and surgical treatment, which suggests more aggressive management. These findings emphasize the potential value of earlier detection strategies to reduce treatment burden and improve outcomes in younger adults. They also underscore the need to address diagnostic delays that may affect this population across geographic settings.

## Comparison to prior research

Our findings build upon a growing body of research documenting the increasing burden of EO-CRC and the geographic disparities in cancer outcomes [19–21]. While prior studies have emphasized poorer outcomes and delayed diagnoses among rural populations, our results suggest that age may be a more dominant factor than rurality in predicting late-stage diagnosis. Specifically, patients aged 30 to 49 had consistently higher odds of presenting with late-stage CRC regardless of geographic residence. This finding highlights a potential shift in the risk profile of EO-CRC, emphasizing that younger adults across both rural and non-rural settings are vulnerable to diagnostic delays [22]. Existing literature has proposed that rural patients may experience asymptomatic disease, misinterpret symptoms such as rectal bleeding, or face provider delays due to misdiagnosis or inadequate physical exams [23, 24], our joint age–rural analyses did not reveal large rural, non-rural differences in late-stage diagnosis among younger adults. However, rural residence remained independently associated with higher CRC-specific mortality after accounting for competing risks, suggesting that survival disparities extend beyond stage at diagnosis alone.

Accounting for competing risks of non-CRC mortality, rurality remained associated with increased CRC-specific death, suggesting that survival differences may extend beyond stage at diagnosis. Therefore, targeted early detection strategies may need to extend beyond rural health disparities to address diagnostic barriers affecting younger adults more broadly. Although our findings are generally consistent with previous studies indicating more aggressive treatment patterns in EO-CRC, they also highlight gaps in evidence-based protocols for younger adults [25]. Patients aged 30 to 49 were significantly more likely to receive chemotherapy and radiation, and those aged 20 to 49 had higher odds of undergoing surgery in both rural and non-rural settings. Previous studies suggest that EO-CRC patients are more likely to receive aggressive interventions, including multiagent chemotherapy and surgical resection for both early and metastatic stages [26–28]. However, given the lack of standardized treatment guidelines for EO-CRC, variation in care may reflect provider discretion or uncertainty. The greater likelihood of adjuvant or neoadjuvant therapy observed in EO-CRC patients diagnosed at stage I or II further supports the need for age-specific clinical protocols [29].

## Study strengths and limitations

This study should be interpreted considering several strengths and limitations. Behavioral factors such as physical activity, diet, smoking, and alcohol use were not available, as these variables are not routinely captured in cancer registry data. These behaviors can influence cancer outcomes and vary by geography; for example, rural residents are more likely to be obese, physically inactive, and use tobacco. Although rural adults are more likely to abstain from alcohol, those who do drink are more likely to exceed recommended limits [30]. The absence of these variables limits our ability to assess lifestyle-related risk. Additionally, comorbidities were not included, which may affect comparisons across age groups.

Socioeconomic status, which plays an important role in both prevention and treatment access, was also not captured in the dataset. We also did not account for competing risks of mortality, particularly relevant among older adults, which may affect the interpretation of CRC-specific survival estimates. Crucially, competing risks of mortality represent a key concern in CRC survival analyses, particularly among older adults who face higher non-CRC mortality. To address this limitation, we conducted stratified Fine–Gray competing-risk models that explicitly accounted for deaths from other causes and incorporated diagnosis year to reflect temporal changes in screening and treatment practices. These additional analyses reduce bias in CRC-specific survival estimates and strengthen inference regarding age-related and rural, non-rural disparities.

Potential selection bias should also be contemplated. Our analytic cohort excluded individuals younger than 20 years or older than 79 years, as well as those with missing or unknown rural, non-rural residence. While these exclusions were necessary for analytic consistency, they may limit the generalizability of our findings to pediatric, geriatric, or geographically unclassified populations. In addition, patients with unknown stage were included in the cohort but excluded from late-stage diagnosis models, which could introduce bias if stage missingness is associated with disease severity. These factors should be considered when interpreting associations involving stage at diagnosis and survival.

Limitations in treatment data must also be acknowledged. Although our secondary analyses showed younger patients had higher odds of receiving chemotherapy, radiation, and surgery, SEER may underreport treatment details, leading to underestimation of care patterns. Moreover, we could not evaluate structural barriers in rural areas, such as transportation and provider shortages. Despite these limitations, the study has important strengths. It is among the first to examine age-related differences in EO-CRC outcomes using a nationally representative dataset, while also accounting for geographic residence. Although rurality did not appear to significantly modify late-stage diagnosis or mortality risk, our stratified analysis adds important nuance to existing literature. These findings highlight the broader need for targeted prevention and intervention strategies that extend beyond rural, non-rural differences. Future research should incorporate more comprehensive data to examine behavioral, clinical, and structural drivers of EO-CRC outcomes.

## Implications for practice and policy

Our results highlight the demand for tailored approaches to EO-CRC prevention, detection, and management. Given the rising incidence of CRC among adults under 50, particularly those aged 30 to 49, current screening thresholds may be inadequate. While lowering the screening age to 45 was a critical step, many younger adults remain ineligible, despite presenting with symptoms. Clinicians, especially in rural areas, must remain attentive in recognizing early signs of CRC and avoid dismissing symptoms in younger patients. The observed treatment patterns suggest that younger patients often receive aggressive care, yet the lack of formal guidelines for EO-CRC may lead to inconsistent treatment decisions. These findings can inform the development of age-specific guidelines and support broader policy efforts aimed at improving early detection and access to care for underserved populations. Public health strategies should also focus on increasing awareness of EO-CRC symptoms among younger adults and enhancing provider training, particularly in settings where access and diagnostic delays persist.

Future research should evaluate the feasibility and effectiveness of lowering the age of screening eligibility and develop targeted tools to identify younger individuals at risk. Studies that incorporate behavioral, clinical, and socioeconomic data will be important for understanding the full range of factors influencing EO-CRC outcomes. Addressing the intersection of age, geography, and access to care is essential to improving early detection and reducing the rising burden of EO-CRC across diverse populations. Results may also support the development of risk assessment tools or symptom indices for identifying individuals at higher risk for EO-CRC. Additionally, improving patient and provider communication around treatment preferences, potential side effects, and access to financial assistance resources may contribute to a better quality of life and more patient-centered care.

## Conclusions

This study highlights the complex relationship between age, rurality, and EO-CRC outcomes. While younger adults, particularly those aged 20 to 29, experience a lower risk of late-stage diagnosis and CRC-specific mortality, older adults face significantly worse outcomes, especially in non-rural areas. The interaction between age and geographic location suggests that structural, clinical, and behavioral factors may shape disparities in diagnosis, treatment, and survival. These findings emphasize the need for age- and geography-sensitive interventions to improve EO-CRC outcomes and reduce the burden of colorectal cancer among younger populations.

## Supplementary Information

Below is the link to the electronic supplementary material.

Supplementary file1 (DOCX 51 KB)

Supplementary file2 (JPG 61 KB)

## Data Availability

No datasets were generated or analysed during the current study.
