# Supplementary material for: Impact of age and rurality on colorectal cancer outcomes in the United States
Source: Cancer Causes Control. 2026 Mar 19;37(4):61. doi: 10.1007/s10552-026-02150-3 (PMC13002674; doi:10.1007/s10552-026-02150-3)
Supplement: Supplementary file 1 — Supplementary file1 (DOCX 51 KB) [file 10552_2026_2150_MOESM1_ESM.docx]

| **Supplemental Table 1: Odds of Chemotherapy Treatment for CRC Onset b**y **Age Categories, Overall a**nd **Stratified by Rural, Non-Rural Status. SEER Patients, 2006 – 2020.** | | | | |
| --- | --- | --- | --- | --- |
|  | **No. (%) of chemotherapy treatment cases** | **No. of total cases** | **Unadjusted OR (95% CI)** | **Adjusted OR (95% CI)** |
| **Overall** |  | | |  |
| Aged 20 – 29 years | 2,457 (40.7%) | 6,038 | 0.82 (0.78, 0.86) | 0.93 (0.88, 0.99) |
| Aged 30 – 39 years | 11,692 (55.5%) | 21,069 | 1.49 (1.44, 1.53) | 1.38 (1.34, 1.43) |
| Aged 40 – 49 years | 37,803 (55.7%) | 67,856 | 1.49 (1.47, 1.53) | 1.39 (1.36, 1.42) |
| Aged 50 – 59 years | 75,448 (45.6%) | 165,364 | 1.00 (Referent) | 1.00 (Referent) |
| Aged 60 – 69 years | 84,087 (41%) | 205,027 | 0.83 (0.82, 0.84) | 0.83 (0.82, 0.84) |
| Aged 70 – 79 years | 59,675 (31%) | 192,577 | 0.54 (0.53, 0.54) | 0.54 (0.53, 0.55) |
| **Among Rural Patients** |  | | | |
| Aged 20 – 29 years | 262 (42.2%) | 621 | 0.82 (0.69, 0.96) | 0.91 (0.76, 1.09) |
| Aged 30 – 39 years | 1,218 (55.7%) | 2,187 | 1.41 (1.29, 1.54) | 1.35 (1.22, 1.49) |
| Aged 40 – 49 years | 4,292 (55.9%) | 7,666 | 1.43 (1.36, 1.51) | 1.34 (1.26, 1.42) |
| Aged 50 – 59 years | 9,898 (47.1%) | 21,011 | 1.00 (Referent) | 1.00 (Referent) |
| Aged 60 – 69 years | 12,107 (40.6%) | 29,787 | 0.77 (0.74, 0.79) | 0.79 (0.76, 0.82) |
| Aged 70 – 79 years | 9,003 (307%) | 29,285 | 0.49 (0.48, 0.52) | 0.53 (0.50, 0.55) |
| **Among Non-Rural Patients** |  | | | |
| Aged 20 – 29 years | 2,195 (40.5%) | 5,417 | 0.82 (0.78, 0.87) | 0.93 (0.88, 0.99) |
| Aged 30 – 39 years | 10,474 (55.5%) | 18,882 | 1.49 (1.45, 1.54) | 1.39 (1.34, 1.44) |
| Aged 40 – 49 years | 33,511 (55.7%) | 60,190 | 1.51 (1.48, 1.54) | 1.39 (1.36 1.43) |
| Aged 50 – 59 years | 65,550 (45.4%) | 144,353 | 1.00 (Referent) | 1.00 (Referent) |
| Aged 60 – 69 years | 71,980 (41.1%) | 175,240 | 0.84 (0.83, 0.85) | 0.83 (0.82, 0.85) |
| Aged 70 – 79 years | 50,672 (31.0%) | 163,292 | 0.54 (0.53, 0.55) | 0.54 (0.53, 0.55) |
| **P value for multiplicative interaction** | | | <0.0001 | <0.0001 |
| Models adjusted for age, race, marital status, sex, rurality*, stage, primary site, persistent poverty, and diagnosis year.  *Stratified models are not adjusted for rurality. | | | | |

| **Supplemental Table 2: Odds of Radiation Treatment for CRC Onset b**y **Age Categories, Overall a**nd **Stratified by Rural, Non-Rural Status. SEER Patients, 2006 – 2020.** | | | | |
| --- | --- | --- | --- | --- |
|  | **No. (%) of radiation treatment cases** | **No. of total cases** | **Unadjusted OR (95% CI)** | **Adjusted OR (95% CI)^2^** |
| **Overall** |  | | |  |
| Aged 20 – 29 years | 57 (0.67%) | 4,250 | 0.69 (0.63, 0.75) | 0.92 (0.84, 1.02) |
| Aged 30 – 39 years | 316 (3.6%) | 13,026 | 1.13 (1.08, 1.17) | 1.16 (1.11, 1.21) |
| Aged 40 – 49 years | 1,002 (11.5%) | 40,815 | 1.26 (1.23, 1.29) | 1.21 (1.17, 1.24) |
| Aged 50 – 59 years | 1,992 (22.9%) | 101,422 | 1.00 (Referent) | 1.00 (Referent) |
| Aged 60 – 69 years | 2,319 (26.7%) | 139,886 | 0.83 (0.81, 0.85) | 0.92 (0.90, 0.94) |
| Aged 70 – 79 years | 1,844 (21.2%) | 144,801 | 0.59 (0.57, 0.60) | 0.73 (0.71, 0.74) |
| **Among Rural Patients** |  | | |  |
| Aged 20 – 29 years | 6 (0.47%) | 425 | 0.75 (0.59, 0.96) | 0.95 (0.72, 1.25) |
| Aged 30 – 39 years | 35 (2.8%) | 1,332 | 1.09 (0.96, 1.23) | 1.11 (0.97, 1.26) |
| Aged 40 – 49 years | 139 (10.9%) | 4,635 | 1.26 (1.17, 1.35) | 1.20 (1.11, 1.29) |
| Aged 50 – 59 years | 289 (22.7%) | 13,001 | 1.00 (Referent) | 1.00 (Referent) |
| Aged 60 – 69 years | 386 (30.4%) | 20,325 | 0.79 (0.75, 0.83) | 0.88 (0.83, 0.93) |
| Aged 70 – 79 years | 261 (20.5%) | 22,050 | 0.52 (0.49, 0.55) | 0.68 (0.64, 0.72) |
| **Among Non-Rural Patients** |  | | |  |
| Aged 20 – 29 years | 51 (0.69%) | 3,825 | 0.68 (0.63, 0.75) | 0.92 (0.83, 1.02) |
| Aged 30 – 39 years | 281 (3.8%) | 11,694 | 1.14 (1.09, 1.19) | 1.16 (1.11, 1.22) |
| Aged 40 – 49 years | 863 (11.6%) | 36,180 | 1.27 (1.23, 1.30) | 1.21 (1.17, 1.24) |
| Aged 50 – 59 years | 1,703 (22.9%) | 88,421 | 1.00 (Referent) | 1.00 (Referent) |
| Aged 60 – 69 years | 1,933 (26.0%) | 119,561 | 0.84 (0.82, 0.85) | 0.93 (0.91, 0.95) |
| Aged 70 – 79 years | 1,583 (21.3%) | 122,751 | 0.59 (0.58, 0.61) | 0.74 (0.72, 0.75) |
| **P value for multiplicative interaction** | | | <0.0001 | 0.0002 |
| Models adjusted for age, race, marital status, sex, rurality*, stage, primary site, persistent poverty, and diagnosis year.  *Stratified models are not adjusted for rurality. | | | | |

| **Supplemental Table 3: Odds of Surgical Treatment for CRC Onset b**y **Age Categories, Overall a**nd **Stratified by Rural, Non-Rural Status. SEER Patients, 2006 – 2020.** | | | | |
| --- | --- | --- | --- | --- |
|  | **No. (%) of surgical treatment cases** | **No. of total cases** | **Unadjusted OR (95% CI)** | **Adjusted OR (95% CI)** |
| **Overall** |  | | |  |
| Aged 20 – 29 years | 5,081 (84.2%) | 6,038 | 1.23 (1.15, 1.32) | 1.16 (1.07 ,1.26) |
| Aged 30 – 39 years | 17,134 (81.3%) | 21,069 | 1.01 (0.97, 1.05) | 1.06 (1.02, 1.11) |
| Aged 40 – 49 years | 54,711 (80.6%) | 67,856 | 0.97 (0.95, 0.99) | 1.03 (1.00, 1.06) |
| Aged 50 – 59 years | 134,208 (81.2%) | 165,364 | 1.00 (Referent) | 1.00 (Referent) |
| Aged 60 – 69 years | 164,682 (80.3%) | 205,027 | 0.95 (0.93, 0.96) | 0.96 (0.94, 0.98) |
| Aged 70 – 79 years | 154,313 (80.1%) | 192,577 | 0.94 (0.92, 0.95) | 0.93 (0.91, 0.95) |
| **Among Rural Patients** |  | | |  |
| Aged 20 – 29 years | 522 (84.1%) | 621 | 0.75 (0.59, 0.96) | 1.17 (0.90, 1.53) |
| Aged 30 – 39 years | 1,773 (81.1%) | 2,187 | 1.09 (0.96, 1.23) | 0.96 (0.84, 1.10) |
| Aged 40 – 49 years | 6,151 (80.2%) | 7,666 | 1.26 (1.17, 1.35) | 0.97 (0.90, 1.05) |
| Aged 50 – 59 years | 17,039 (81.1%) | 21,011 | 1.00 (Referent) | 1.00 (Referent) |
| Aged 60 – 69 years | 23,914 (80.3%) | 29,787 | 0.79 (0.75, 0.83) | 0.94 (0.89, 0.99) |
| Aged 70 – 79 years | 23,442 (80%) | 29,285 | 0.52 (0.49, 0.55) | 0.94 (0.89, 0.99) |
| **Among Non-Rural Patients** |  | | |  |
| Aged 20 – 29 years | 4,559 (84.2%) | 5,417 | 0.68 (0.63, 0.75) | 1.16 (1.06, 1.26) |
| Aged 30 – 39 years | 15,361 (81.4%) | 18,882 | 1.14 (1.09, 1.19) | 1.08 (1.03, 1.13) |
| Aged 40 – 49 years | 48,560 (80.7%) | 60,190 | 1.27 (1.23, 1.30) | 1.04 (1.01, 1.07) |
| Aged 50 – 59 years | 117,169 (81.2%) | 144,353 | 1.00 (Referent) | 1.00 (Referent) |
| Aged 60 – 69 years | 140,768 (80.3%) | 175,240 | 0.84 (0.82, 0.85) | 0.96 (0.94, 0.98) |
| Aged 70 – 79 years | 130,871 (80.1%) | 163,292 | 0.59 (0.58, 0.61) | 0.93 (0.91, 0.95) |
| **P value for multiplicative interaction** | | | 0.47 | 0.03 |
| Models adjusted for age, race, marital status, sex, rurality*, stage, primary site, persistent poverty, and diagnosis year.  *Stratified models are not adjusted for rurality. | | | | |

| **Supplemental Table 4:**  **Fine–Gray Proportional Sub-Distribution Hazard Models for Colorectal Cancer–Specific Mortality, Stratified by Age and Accounting for Competing Risks. SEER Patients, 2006 – 2020.** | | | | | | |
| --- | --- | --- | --- | --- | --- | --- |
| **Age Groups** | **No. (%) of CRC deaths** | **No. (%) of Competing deaths** | **Mean Survival Months (SE)** | **Sub-Distribution Hazard Ratio (SHR) and 95% Confidence Interval (CI)** | | |
|  | | | | **Model 1** | **Model 2** | **Model 3** |
| **Aged 20-29 years** | 1,232 (20.4%) | 205 (3.4%) |  | | | |
| Rural | 141 (22.7%) | 24 (3.9%) | 55.2 (1.94) | 1.11 (0.93, 1.33) | 1.19 (0.99, 1.43) | **1.21 (1.02, 1.45)** |
| Non-Rural | 1,091 (20.1%) | 181 (3.3%) | 55.2 (0.65) | 1.00 (Referent) | 1.00 (Referent) | 1.00 (Referent) |
| **Aged 30-39 years** | 5,045 (23.9%) | 992 (4.7%) |  | | | |
| Rural | 544 (10.5%) | 127 (5.8%) | 56.3 (1.06) | 1.05 (0.96, 1.15) | **1.10 (1.01, 1.21)** | **1.15 (1.05, 1.26)** |
| Non-Rural | 4,501 (23.9%) | 865 (4.6%) | 57.7 (0.36) | 1.00 (Referent) | 1.00 (Referent) | 1.00 (Referent) |
| **Aged 40-49 years** | 18,005 (26.5%) | 4,294 (6.3%) |  | | | |
| Rural | 2,212 (28.9%) | 570 (7.4%) | 60.1 (0.58) | **1.09 (1.05, 1.15)** | **1.11 (1.07, 1.17)** | **1.13 (1.08, 1.19)** |
| Non-Rural | 15,793 (26.2%) | 3,724 (6.2%) | 61.2 (0.21) | 1.00 (Referent) | 1.00 (Referent) | 1.00 (Referent) |
| **Aged 50-59 years** | 40,330 (24.4%) | 15,464 (9.4%) |  | | | |
| Rural | 5,733 (27.3%) | 2,360 (11.2%) | 60.5 (0.35) | **1.14 (1.11, 1.17)** | **1.13 (1.10, 1.17)** | **1.12 (1.08, 1.15)** |
| Non-Rural | 34,597 (23.9%) | 13,104 (9.1%) | 62.0 (0.13) | 1.00 (Referent) | 1.00 (Referent) | 1.00 (Referent) |
| **Aged 60-69 years** | 53,950 (26.3%) | 32,724 (16.0%) |  | | | |
| Rural | 8,264 (27.7%) | 5,341 (17.9%) | 55.3 (0.29) | **1.07 (1.04, 1.09)** | **1.07 (1.04, 1.09)** | **1.08 (1.06, 1.11)** |
| Non-Rural | 45,686 (26.1%) | 27,383 (15.6%) | 56.6 (0.12) | 1.00 (Referent) | 1.00 (Referent) | 1.00 (Referent) |
| **Aged 70-79 years** | 54,854 (28.5%) | 53,296 (27.7%) |  | | | |
| Rural | 8,705 (29.7%) | 8,501 (29.0%) | 48.2 (0.27) | **1.06 (1.03, 1.08)** | **1.08 (1.05, 1.11)** | **1.08 (1.05, 1.10)** |
| Non-Rural | 46,149 (28.3%) | 44,795 (27.4%) | 49.9 (0.12) | 1.00 (Referent) | 1.00 (Referent) | 1.00 (Referent) |
| **Model 1:** Adjusted for rurality and diagnosis years.  **Model 2**: Adjusted for rurality, diagnosis years, race, marital status, sex, and persistent poverty.  **Model 3:** Adjusted for rurality, diagnosis years, race, primary site, stage, marital status, sex, persistent poverty, surgery, radiation, and chemotherapy. | | | | | | |

| Supplemental Table 5: Joint Association of Age Group and Rurality With Late-Stage Diagnosis, CRC-Specific Mortality, and Mean Survival. SEER Patients, 2006-2020. | | | | | | |
| --- | --- | --- | --- | --- | --- | --- |
|  | | | | **Odds Ratio (OR) and 95% Confidence Interval (CI)** | | |
| **Age Group** | **Mean Survival Months (SE)** | **No. Late-Stage Diagnosis (%)** | **No. Obs** | **Model 1** | **Model 2** | **Model 3** |
| **Aged 20-29 years** |  | | | | | |
| Rural | 55.25 (1.94) | 247 (39.8%) | 621 | 0.93 (0.77, 1.13) | 0.90 (0.75, 1.09) | 0.81 (0.65, 1.01) |
| Non-Rural | 55.19 (0.65) | 2,058 (37.9%) | 5,417 | 0.90 (0.84, 0.96) | 0.85 (0.79, 0.91) | 0.78 (0.72, 0.85) |
| **Aged 30-39 years** |  | | | | | |
| Rural | 56.33 (1.06) | 1,047 (47.9%) | 2,187 | 1.42 (1.28, 1.58) | 1.43 (1.28, 1.58) | 1.12 (0.99, 1.27) |
| Non-Rural | 57.66 (0.36) | 9,094 (48.2%) | 18,882 | 1.54 (1.48, 1.60) | 1.52 (1.46, 1.58) | 1.25 (1.19, 1.31) |
| **Aged 40-49 years** |  | | | | | |
| Rural | 60.10 (0.58) | 3,818 (49.8%) | 7,666 | 1.43 (1.35, 1.51) | 1.45 (1.37, 1.53) | 1.21 (1.13, 1.29) |
| Non-Rural | 61.17 (0.21) | 29,875 (49.6%) | 60,190 | 1.44 (1.41, 1.47) | 1.44 (1.41, 1.47) | 1.22 (1.19, 1.26) |
| **Aged 50-59 years** |  | | | | | |
| Rural | 60.49 (0.35) | 9,540 (45.4%) | 21,011 | 1.07 (1.04, 1.11) | 1.09 (1.05, 1.12) | 1.07 (1.03, 1.11) |
| Non-Rural | 62.03 (0.13) | 62,795 (43.5%) | 144,353 | Referent | Referent | Referent |
| **Aged 60-69 years** |  | | | | | |
| Rural | 55.25 (0.29) | 12,967 (43.5%) | 29,787 | 1.01 (0.98, 1.04) | 1.03 (0.99, 1.06) | 1.13 (1.09, 1.17) |
| Non-Rural | 56.55 (0.12) | 76,529 (43.7%) | 175,240 | 1.03 (1.01, 1.04) | 1.02 (1.01, 1.04) | 1.14 (1.11, 1.16) |
| **Aged 70-79 years** |  | | | | | |
| Rural | 48.17 (0.27) | 12,529 (42.8%) | 29,285 | 0.96 (0.93, 0.99) | 0.97 (0.94, 1.00) | 1.33 (1.29, 1.38) |
| Non-Rural | 49.92 (0.12) | 69,723 (42.7%) | 163,292 | 0.95 (0.93, 0.97) | 0.95 (0.93, 0.97) | 1.30 (1.28, 1.33) |
|  | | | | **Sub-Distribution Hazard Ratio (SHR) and 95% Confidence Interval (CI)** | | |
| **Aged 20-29 years** | **Mean Survival Months (SE)** | **No. (%) of Competing deaths** | **No. Obs** | **Model 1** | **Model 2** | **Model 3** |
| Rural | 86.97 (1.98) | 24 (3.86%) | 621 | 1.01 (0.86, 1.19) | 0.98 (0.83, 1.15) | 1.03 (0.89, 1.20) |
| Non-Rural | 124.05 (1.05) | 181 (3.34%) | 5,417 | 0.90 (0.85, 0.96) | 0.86 (0.81, 0.91) | 0.90 (0.85, 0.95) |
| **Aged 30-39 years** |  | | | | | |
| Rural | 102.73 (1.47) | 127 (5.81%) | 2,187 | 1.10 (1.04, 1.19) | 1.12 (1.03, 1.21) | 1.01 (0.94, 1.19) |
| Non-Rural | 119.46 (0.59) | 865 (4.58%) | 18,882 | 1.05 (1.02, 1.08) | 1.05 (1.01, 1.08) | 0.93 (0.90, 0.95) |
| **Aged 40-49 years** |  | | | | | |
| Rural | 110.39 (0.92) | 570 (7.44%) | 7,666 | 1.23 (1.18, 1.28) | 1.24 (1.19, 1.29) | 1.11 (1.06, 1.15) |
| Non-Rural | 118.36 (0.34) | 3,724 (6.19%) | 60,190 | 1.12 (1.10, 1.14) | 1.12 (1.10, 1.14) | 1.00 (0.98, 1.02) |
| **Aged 50-59 years** |  | | | | | |
| Rural | 108.39 (0.56) | 2,360 (11.23%) | 21,011 | 1.14 (1.11, 1.17) | 1.14 (1.11, 1.18) | 1.12 (1.09, 1.15) |
| Non-Rural | 118.56 (0.22) | 13,104 (9.08%) | 144,353 | Referent | Referent | Referent |
| **Aged 60-69 years** |  | | | | | |
| Rural | 96.92 (0.48) | 5,341 (17.93%) | 29,787 | 1.17 (1.15, 1.20) | 1.19 (1.16, 1.22) | 1.22 (1.19, 1.25) |
| Non-Rural | 103.13 (0.20) | 27,383 (15.63%) | 175,240 | 1.10 (1.09, 1.12) | 1.11 (1.09, 1.12) | 1.12 (1.10, 1.13) |
| **Aged 70-79 years** |  | | | | | |
| Rural | 75.75 (0.45) | 8,501 (29.03%) | 29,285 | 1.28 (1.25, 1.31) | 1.30 (1.27, 1.34) | 1.40 (1.37, 1.44) |
| Non-Rural | 80.24 (0.19) | 44,795 (27.43%) | 163,292 | 1.21 (1.19, 1.23) | 1.21 (1.19, 1.23) | 1.30 (1.29, 1.32) |
| **Model 1:** Adjusted for rurality and diagnosis years.  **Model 2:** Adjusted for rurality, diagnosis years, race, marital status, sex, and persistent poverty.  **Model 3:** Adjusted for rurality, diagnosis years, race, primary site, stage, marital status, sex, persistent poverty, surgery, radiation, and chemotherapy. | | | | | | |
