# Supplementary figures and images for: Impact of age and rurality on colorectal cancer outcomes in the United States
Source: Cancer Causes Control. 2026 Mar 19;37(4):61. doi: 10.1007/s10552-026-02150-3 (PMC13002674; doi:10.1007/s10552-026-02150-3)

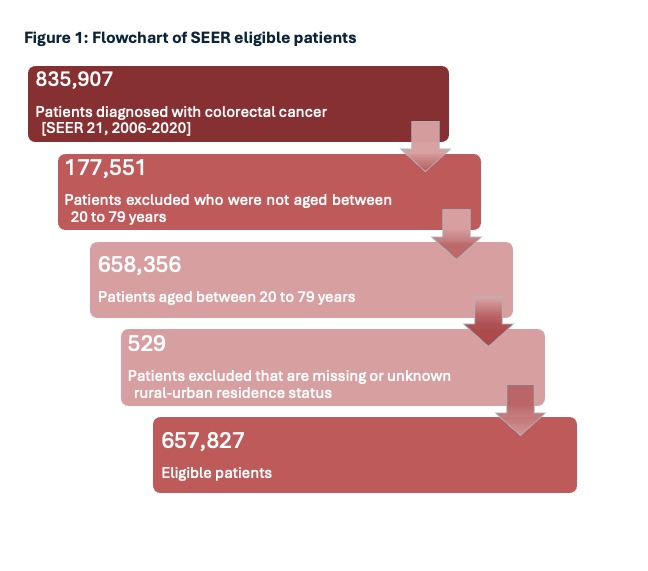

Supplement: Supplementary file 2 — Supplementary file2 (JPG 61 KB) [file 10552_2026_2150_MOESM2_ESM.jpg]
